# Real-Time Rainfall Forecasts Based on Radar Reflectivity during Typhoons: Case Study in Southeastern Taiwan

**DOI:** 10.3390/s21041421

**Published:** 2021-02-18

**Authors:** Chih-Chiang Wei, Chen-Chia Hsu

**Affiliations:** Department of Marine Environmental Informatics & Center of Excellence for Ocean Engineering, National Taiwan Ocean University, Keelung 20224, Taiwan; itriGary8448@itri.org.tw

**Keywords:** typhoon, rainfall, prediction, radar, machine learning

## Abstract

This study developed a real-time rainfall forecasting system that can predict rainfall in a particular area a few hours before a typhoon’s arrival. The reflectivity of nine elevation angles obtained from the volume coverage pattern 21 Doppler radar scanning strategy and ground-weather data of a specific area were used for accurate rainfall prediction. During rainfall prediction and analysis, rainfall retrievals were first performed to select the optimal radar scanning elevation angle for rainfall prediction at the current time. Subsequently, forecasting models were established using a single reflectivity and all elevation angles (10 prediction submodels in total) to jointly predict real-time rainfall and determine the optimal predicted values. This study was conducted in southeastern Taiwan and included three onshore weather stations (Chenggong, Taitung, and Dawu) and one offshore weather station (Lanyu). Radar reflectivities were collected from Hualien weather surveillance radar. The data for a total of 14 typhoons that affected the study area in 2008–2017 were collected. The gated recurrent unit (GRU) neural network was used to establish the forecasting model, and extreme gradient boosting and multiple linear regression were used as the benchmarks. Typhoons Nepartak, Meranti, and Megi were selected for simulation. The results revealed that the input data set merged with weather-station data, and radar reflectivity at the optimal elevation angle yielded optimal results for short-term rainfall forecasting. Moreover, the GRU neural network can obtain accurate predictions 1, 3, and 6 h before typhoon occurrence.

## 1. Introduction

Taiwan is located in the intertropical convergence zone in the western Pacific Ocean with a geographical location of approximately 22° N–25° N and 120° E–122° E. Taiwan is frequently affected by typhoons during summer and autumn that result in heavy precipitation. Although heavy precipitation causes disasters such as flooding, landslides, and debris flows, it is a main source of water [1,2]. Therefore, when typhoons approach, government agencies must make various cautious decisions regarding future strong winds and heavy precipitation, e.g., reservoir discharges. This study developed a system that can forecast future rainfall time patterns and then used them to enact timely typhoon-prevention measures.

Weather radar is an essential analysis tool for meteorological observations because it can be used to locate weather patterns, large-scale rainfall, and airflow characteristics [3,4,5,6]. Radar emits electromagnetic waves from an antenna. When contacting targets in the air (e.g., water vapor), the waves are scattered and partially reflected back to the radar direction [7,8]. Marshall et al. [9] calculated the reflectivity power and raindrop size distribution observed from a disdrometer at a distance of 8.8 km from the radar and demonstrated an excellent correlation with the calculated reflectivity factor (Z). Marshall and Palmer [10] proposed Z = 200 × *R*^1.6^ and explained the relationship between Z and rainfall intensity (R). Studies on convective systems have, however, underestimated quantitative precipitation (e.g., [11,12]). Therefore, these coefficients may not be the most suitable for predicting heavy rainfall during typhoons and should be refined. Studies have been conducted on rainfall retrieval and estimation by using the Z–R relationship [13,14,15,16,17,18,19,20,21]. For instance, Qiu et al. [22] developed radar–rain gauge merging methods to predict precipitation by combing the advantages of rain-gauge observations and radar quantitative precipitation estimation. In addition, weather in Taiwan has been studied [23,24,25]. For example, Ochoa-Rodriguez et al. [26] provided an overview of merging techniques and application-oriented categorization methods, including radar bias-adjustment, rain-gauge interpolation using radar spatial association, and radar–rain gauge integration.

Although weather radars provide excellent real-time monitoring of the weather, with high spatial and temporal resolution [27,28], rainfall estimation has many acquisition limitations, such as a limited radar coverage area because of the complexity of the terrain and the shading problem [25,29] (which will be discussed subsequently). Satellite observations is an alternative to radar observation. With advanced infrared and microwave instruments, satellite observations overcome the aforementioned limitations by providing a spatially homogeneous and temporal coverage for vast areas [30]. Although satellite observations are useful for monitoring mesoscale convective systems, they are inadequate for studying the small-scale internal precipitation structure of mesoscale convective systems, such as typhoons [31]. Nevertheless, they provide critical weather information and facilitate hydrological monitoring [30].

Scofield and Kuligowski [29] revealed that the transformation of the radar-measured reflectivities into rainfall rates has three challenges. The first problem is the accuracy of the reflectivity values, which can be affected by fixed targets (e.g., ground clutter and beam block) or anomalous propagation. Second, the increase in the elevation of the beam from the radar leads to errors associated with uncertainties in the shape of the reflectivity profile between the beam height and the surface; precipitation is typically undetected, or the rate is underestimated, as the distance from the radar increases [32,33,34]. Third, the relationship between reflectivity and the rainfall rate (i.e., the Z–R relationship) is related to the drop size distribution and is also affected by beam spreading [35]. Several studies have addressed these problems [36,37]. This study focused on the first and second problems related to overshooting of the beam and its blockage.

According to the Central Weather Bureau (CWB) of Taiwan [38], radar reflectivity at a specific scan elevation angle does not necessarily reflect the intensity of clouds and rain. For example, when radar pulses are transmitted at a certain elevation angle, the vertical elevation of the radar scan is lower when the observation location is farther from the radar. Therefore, when the vertical top of a rain system is lower than the height of the radar pulses at a certain elevation angle, no reflectivity is returned, but rainfall still occurs at the ground observation station. Second, radar observations are affected by topography that creates an observation barrier, making rainfall conditions behind mountains unobservable. Radar stations in Taiwan, such as the Gematronik METEOR 1000S system with a wavelength of 10 cm (S-band), adopted the scanning strategy of volume coverage pattern 21 (VCP21) to fit the Weather Surveillance Radar-1988 Doppler (WSR-88D) [39]. This strategy involves scans at nine elevation angles (0.5°, 1.4°, 2.4°, 3.4°, 4.3°, 6.0°, 9.9°, 14.6°, and 19.5°) [39,40]. Because VCP21 scanning strategy involves multiple elevation angles, a specific scanning angle may be able to reflect optimal precipitation characteristics through rainfall retrievals.

On the basis of the aforementioned problems, this study developed an hourly conceptual system for rainfall forecasting during typhoons based on radar reflectivity. This system can be used to address the following problems: (1) identifying the most suitable elevation angle or combination of elevation angles from weather surveillance radar (WSR) scans for real-time ground precipitation forecasting, and (2) evaluating radar reflectivity accuracy in terms of precipitation prediction at various observation locations with terrain blockage.

In the establishment of the rainfall forecasting model, input data included merged radar reflectivity data and meteorological data from ground-weather stations. Conventionally, linear regression models can provide a prediction model on the basis of statistical theory and reasonable results. Therefore, researchers have used linear and nonlinear regression models to develop rainfall forecast or prediction models [41,42,43]. Furthermore, the application of ensembles in numerical weather prediction improved weather forecasts [44,45,46]. For example, Shakti et al. [47] used the short-term ensemble prediction system to generate ensemble nowcasts of rainfall. The ensemble nowcasts were verified using radar rainfall data.

Because of the rapid growth of data collected using various sensors (e.g., satellites, radar remote sensors, and meteorological instruments), characteristic data available for model input have increased substantially when establishing forecasting models. Consequently, mathematical models, e.g., machine learning (ML)-based models that can account for high dimensions and nonlinearity, are required to improve prediction accuracy. Numerous ML models have been successfully applied in related research on rainfall retrievals and estimations, such as [48,49,50,51,52,53,54]. XGB is a popular ML algorithm developed by Chen and Guestrin [55]. XGB is effective because it is a scalable ML system for tree boosting. XGB scalability is determined by the optimization of vital algorithms, including a new tree learning algorithm for processing sparse data and a weighted quantile sketch process. XGB can simplify learning through models and prevent overfitting; therefore, its calculative abilities are superior to those of traditional gradient-boosted decision trees [56]. Therefore, XGB has been used by various authors such as Chakraborty and Alajali [57] and Yuan et al. [58]. Wei and Hsu [59] addressed the rainfall retrieval problem for quantitative precipitation estimation. The feasibility of rain retrievals was examined from linear regressions, support vector regressions, and XGB models. However, their models did not have memory functions and could not handle sequence dependence; limited time-series data were available for use and could result in inaccuracy.

Although various ML models have been developed, accurate forecasting precipitation remains a challenge. However, recurrent neural networks (RNNs) exhibit promising results in time-series data [60]. RNNs are deep-learning models that have been used extensively for time sequence data [61]. Numerous RNNs, such as the long short-term memory (LSTM) network [62], have been developed. The LSTM network can address the gradient-vanishing problem by introducing a gate control unit, and LSTM has been widely used in the field of time-series data prediction [63,64]. Cho et al. [65] proposed a LSTM-based network gated recurrent unit (GRU) model, which learns how to use its gates to protect its memory to optimize the network structure and make long-term predictions [66]. The GRU can achieve performance comparable to LSTM and maintain the prediction effect with fewer training parameters than LSTM can [61]. Misra et al. [67] used the LSTM model to capture the spatiotemporal dependencies in local rainfall. Kumar et al. [68] used LSTM for forecasting monthly rainfall by using long sequential raw data for time-series analysis. Chhetri et al. [69] presented a GRU-based model for rainfall prediction using weather parameters (temperature, rainfall, relative humidity, sunshine hour, and wind speed). Although advanced GRUs exhibit advantages in developing forecasting models, they have seldom been used in rainfall forecasting using radar reflectivities.

This study used ML-based models to establish typhoon rain prediction models. The GRU algorithm was adopted in the present study to improve the accuracy of rainfall prediction, and XGB and conventional multiple linear regression (MLR) were used as benchmark models for comparison.

## 2. Data Description

**Figure 1:**
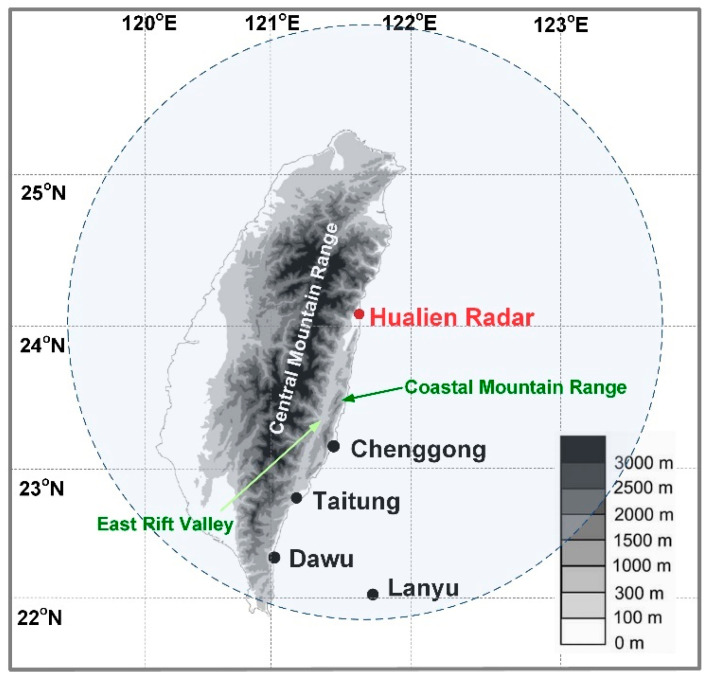
Locations of the Hualien WSR and experimental stations.

**Figure 2:**
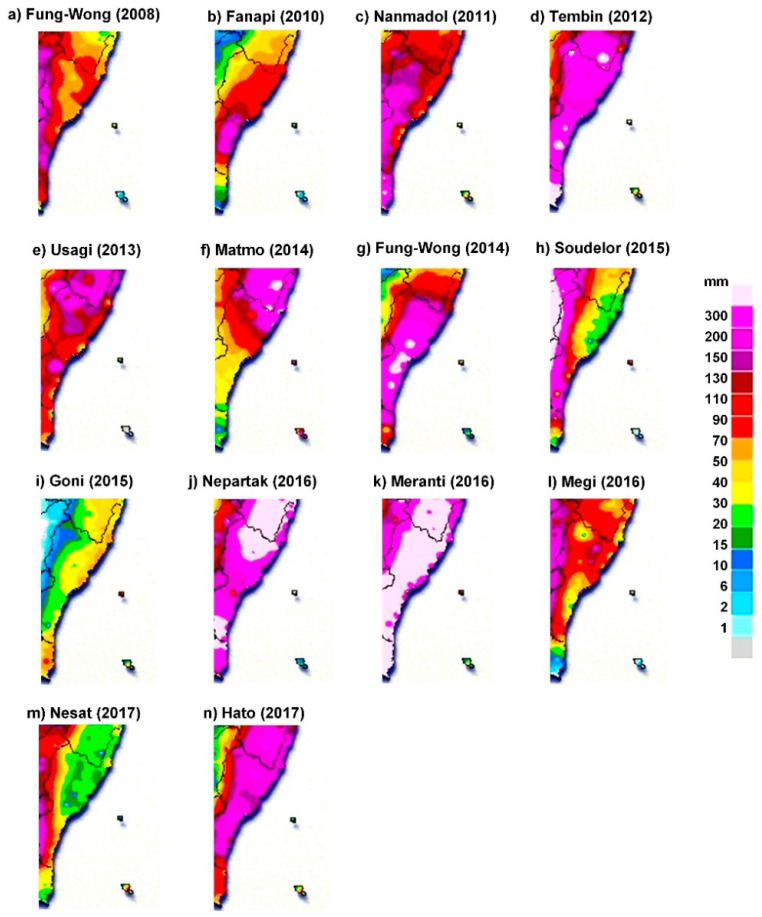
Spatial distribution of 24-h accumulated rainfall over the study area: (**a**) Fung-Wong (2008); (**b**) Fanapi (2010); (**c**) Nanmadol (2011); (**d**) Tembin (2012); (**e**) Usagi (2013); (**f**) Matmo (2014); (**g**) Fung-Wong (2014); (**h**) Soudelor (2015); (**i**) Goni (2015); (**j**) Nepartak (2016); (**k**) Meranti (2016); (**l**) Megi (2016); (**m**) Nesat (2017); (**n**) Hato (2017).

The study area (Figure 1) was southeastern Taiwan and included three onshore weather stations (Chenggong, Taitung, and Dawu stations) and one offshore station (Lanyu station). The selected experimental areas are located in typical typhoon paths and are affected by typhoons. Table 1 lists 14 typhoon events that affected the study area from 2008 to 2017 and their occurrence dates, total precipitation, and maximum wind speeds at the typhoon centers. Moreover, Figure 2 illustrates the spatial distribution of 24-h accumulated rainfall over the study area of each collected typhoon.

This study collected radar reflectivity and ground-weather data from the CWB of Taiwan. The CWB directly supervises four weather radar stations [38], which comprise a Doppler weather radar network in Taiwan. Hualien Weather Radar Station (Figure 1) was selected as the experimental radar station because Hualien Weather Radar Station covers the experimental area and is not directly blocked by Central Mountain Range (although slightly blocked by Coastal Mountain Range and East Rift Valley). The Hualien Weather Radar Station was established in 2001 and uses the METEOR 1000S radar model, which is an S-band (wavelength of 10 cm) Doppler weather radar. The radar can continually provide data of three high-precision and high-resolution measurements, namely reflectivity, mean radial velocity, and spectral width within 5–10 min [38]. Its geographical coordinates are 23.99° N, 121.62° E, and it has an elevation of 63 m and observation radius of 460 km. The radar can be used to observe weather systems such as typhoons and rainy seasons from the Pacific Ocean, South China Sea, and Bashi Channel. When scanning over eastern Taiwan, the Hualien WSR uses true north as the starting point to perform a 360-degree clockwise conical surface scan, and conical surface data at different elevation angles obtained from each observation can form a complete volume scan. The Hualien WSR uses VCP21 (nine elevation angles) for typhoon and precipitation observations, and a total volume scan can be completed in approximately 6 min [38].

**Figure 3:**
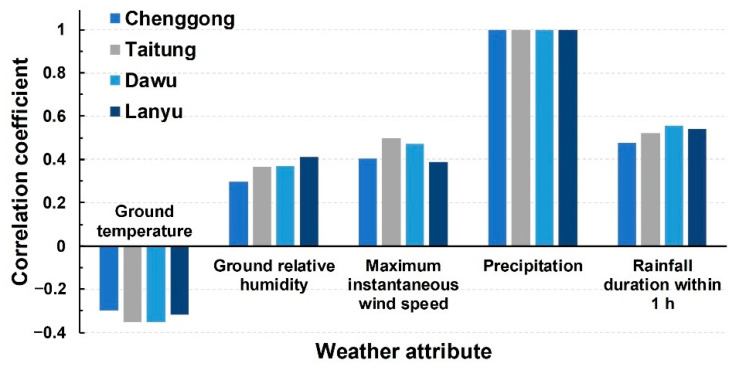
Correlation coefficients of ground-weather and precipitation characteristics at four stations.

Regarding meteorological data, this study collected ground-weather characteristics at four stations. Each station has 12 characteristics, including ground air pressure (hPa), air pressure at sea level (hPa), ground temperature (°C), ground dew-point temperature (°C), ground relative humidity (%), ground global solar radiation (MJ/m^2^), ground vapor pressure (hPa), surface wind speed (m/s), surface wind direction (deg), maximum instantaneous wind speed (m/s), instantaneous wind direction (deg), and precipitation (mm/h). A single station can collect 2544 pieces of hourly records. This study used a correlation coefficient greater than 0.3 (i.e., at least a moderate correlation) between characteristics and precipitation as the characteristic screening threshold, and all four stations were screened simultaneously. In addition to the target precipitation characteristics, four other weather characteristics were selected (i.e., ground temperature, ground relative humidity, maximum instantaneous wind speed, and rainfall duration within 1 h) on the basis of the analysis results. The correlation coefficients of these characteristics at the four stations are presented in Figure 3.

## 3. Prediction System and Models

**Figure 4:**
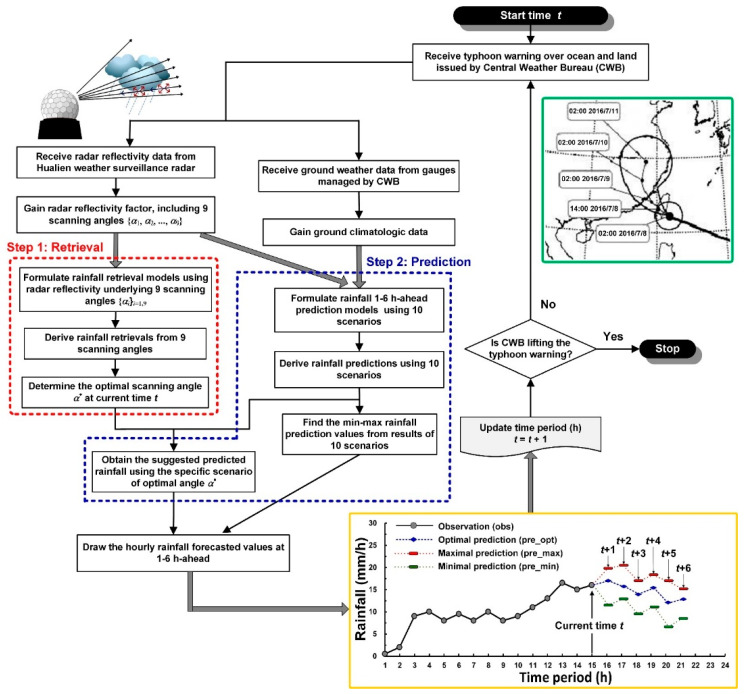
A real-time rainfall forecasting system during typhoons.

Figure 4 presents a schematic of the real-time typhoon rainfall forecasting system developed in this study. After the CWB issued a typhoon warning over ocean and land, the system collected radar reflectivity and ground-weather data in the area. This process of rainfall prediction is performed in two steps, namely precipitation retrieval (step 1, dashed red box in Figure 4) and precipitation prediction (step 2, dashed blue box). Before the precipitation forecast model is established, rainfall retrieval is performed to determine the optimal radar elevation angle for precipitation prediction at current time. Rainfall retrieval is performed using the reflectivity values {*α_i_*}*_i_*_=1,9_ of the nine elevation angles using VCP21 scanning strategy to develop the retrieval values at the current time. This study used the relationship *Z* = a × *R*^b^, and the parameters a and b were derived depending on the elevation angle because the nine elevation angles, nine Z–R relations were established at each station. Thus, the retrieval values of the individual radar elevation angles of the four stations could be inferred separately.

The Z–R relations were used only to determine the optimal elevation angle α*. However, this relation may underestimate the quantitative precipitation estimation on convective systems [40]. The absolute values of the differences between the retrieval values and the observation values (assuming that the observed rainfall at the current time was obtained in real time) at the current time are subsequently compared, and the angle corresponding to the least absolute errors is the optimal scanning angle α* at the current time.

The second step is precipitation prediction. The input data of the precipitation prediction model include data sets {**Z**}, {**W**}, and {**R**}. Here, the reflectivity characteristics of nine elevation angles in the VCP21 system were expressed as data set {**Z**} = {*Z_i_*}*_i_*_=1,9_; the four ground-weather characteristics are represented as data set {**W**}; and the precipitation characteristics are represented as data set {**R**}. Then, the precipitation prediction *P_t_*_+__Δ*t*_ can be expressed as

*P_t_*_+__Δ*t*_ = *f* ({**Z,W,R**}) (1)

where *t* is the current time, and Δ*t* is the forecast horizon (the forecast horizons in this study were 1, 3, and 6 h).

To design the prediction scenarios, this study established a single reflectivity prediction model (scenarios 1–9) from the nine elevation angles of VCP21. For example, scenario 1 is *P_t_*_+__Δ*t*_ = *f* ({*Z*_1_,**W**,**R**}), and *Z*_1_ = radar reflectivity at 0.5°. In addition, this study added a scenario in which all elevation angles {*α*_1_, *α*_2_, *…*, *α*_9_} were the input characteristics (scenario 10). Therefore, when the system forecasts rainfall in real time, 10 sets of predicted values are generated.

Of the 10 sets of generated prediction values, the scenario corresponding to the optimal elevation angle α* in rainfall retrieval at the current time was adopted as the optimal prediction (pred_opt). Moreover, all the maximal predictions (pred_max) and minimal predictions (pred_min) of the scenarios were used to obtain the possible range of pred_max and pred_min.

In this study, GRU and XGB were used to establish the rainfall forecasting model. The GRU model, a variant of the LSTM neural network, is composed of the input, forget, and output layers [63]. The input gate regulates the amount of information that enters the memory cell, the forget gate directs the memory cell and remains in the present memory cell through recurring connection, and the output gate determines the amount of data used to calculate the output activation of the memory cell and information flow to the rest of the neural network [60]. Similar to the LSTM unit, the GRU has gating units that modulate the flow of information inside the unit without having a separate memory cell [70]. The weights corresponding to these gates are updated through backpropagation through time stochastic gradient descent to minimize a loss function [62,71]. The GRU algorithm was derived by Cho et al. [65] and Chung et al. [71].

XGB is a type of tree-based boosting method that is a sequential ensemble learning model of a decision tree [72]. The principal feature of XGB is its ability to automatically use multithreading for parallel computing while improving algorithm accuracy. XGB can perform parallel tree boosting (also known as gradient-boosted decision trees), which rapidly and accurately solves numerous data problems [73]. XGB uses a second-order Taylor expansion for the loss function and uses both the first- and second-order derivatives. To avoid overfitting, the regularization term is added to help smooth the final learned weights. See Chen and Guestrin [55] and Jia et al. [74] for details on algorithm derivation in XGB.

## 4. Modeling and Verification

In this study, three typhoon events in 2016, namely Typhoons Nepartak, Meranti, and Megi, were selected to be test events. The remaining 11 typhoons were used as training and validation data sets. The rainfall forecasting model necessitated a test for optimal rainfall lag time because precipitation was the predicted target characteristic of the model. The test was also required to increase effective information input for improving precipitation prediction accuracy. The training and validation process involved 10-fold cross-validation in which 90% of the data were randomly selected each time as training data, and the remaining 10% were validation data that were not repeatedly selected until all data had been selected as the 10% test set.

This study used root mean squared error (RMSE) to evaluate the errors. RMSE is defined as follows:

(2) $$\mathrm{RMSE}=\sqrt{\frac{1}{n}\overset{n}{\underset{i=1}{\sum }}{\left(p_{i}^{\mathrm{pre}}-p_{i}^{\mathrm{obs}}\right)}^{2}}$$

where *n* is the number of pieces of data; $p_{i}^{\mathrm{pre}}$ is the *i*^th^ predicted value; and $p_{i}^{\mathrm{obs}}$ is the *i*^th^ observed value.

**Figure 5:**
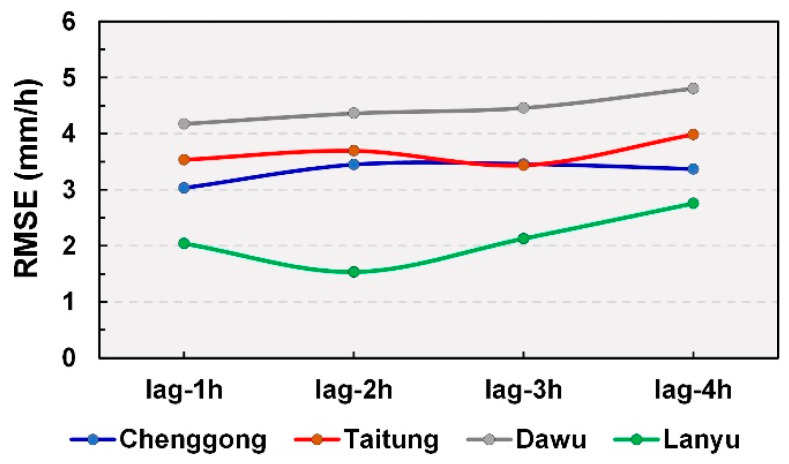
RMSE results at different lag times for each station.

Figure 5 illustrates the validation results of lag time = 1–4 h. According to the figure, errors were minimal for the GRU model (by preset parameters) when the lag time was 1, 3, 1, and 2 h for Chenggong, Taitung, Dawu, and Lanyu stations, respectively. Therefore, increased inputs for testing the optimum rainfall lag time were used for the modeling process.

The model hyperparameters were verified subsequently. For the GRU model, the hyperparameters of the number of the hidden layers and the number of neurons in a hidden layer were calibrated. The ReLU activation function, which is defined as the positive part of its argument, that is, *f*(*x*) = *max* (0, *x*), where *x* is the input to a neuron, was used in the hidden layers. In the optimization process, adaptive moment estimation (Adam) was used to learn the learning rate. Adam is a first-order optimization algorithm that can replace the traditional stochastic gradient descent algorithm, and it can adapt the learning rate to the parameters [75]. The number of hidden layers was set to 1–6 for validation, and the validation range of the number of neurons in a hidden layer was 10–100. First, the number of neurons in a hidden layer was fixed to obtain the number of hidden layers with a small RMSE. Next, the optimal parameter for the number of neurons in a hidden layer was validated.

For the XGB model, the hyperparameters were min_child_weight and max_depth. The min_child_weight refers to the minimum sum of the instance weight required in a child and max_depth to preventing overfitting to avoid tree growing very deep. Modeling was performed using preset parameters, namely min_child_weight = 1 and max_depth = 3 (these hyperparameters were tested in the subsequent section for optimal values). The min_child_weight was set to 1, 2, and 3 for validation, and the validation range of max_depth was 3–18. First, min_child_weight was fixed to obtain a parameter with a small RMSE. After obtaining the optimal min_child_weight parameter, the optimal parameter for max_depth was validated.

**Figure 6:**
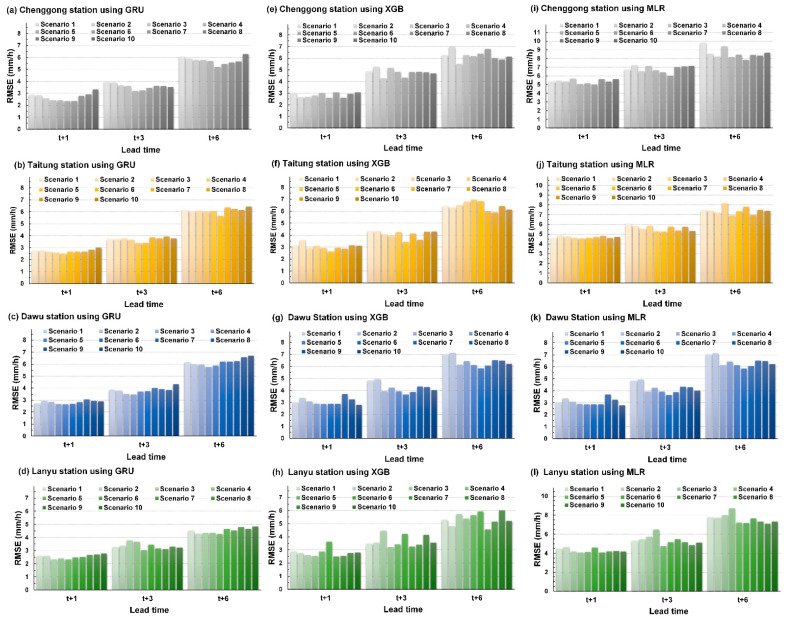
RMSE indices of the forecasting models for each station: (**a**–**d**) GRU for Chenggong, Taitung, Dawu, and Lanyu stations; (**e**–**h**) XGB for Chenggong, Taitung, Dawu, and Lanyu stations; (**i**–**l**) MLR for Chenggong, Taitung, Dawu, and Lanyu stations.

Figure 6 illustrates the validation results of the GRU, XGB, and MLR forecasting models at Chenggong, Taitung, Dawu, and Lanyu stations. The figure plots the RMSE performance of scenarios 1–10 at lead time = 1, 3, and 6 h. For GRU models, Figure 6a–d indicates that the longer the forecast horizon is, the larger the prediction error is. In addition, performance data in each scenario at each station were organized. At lead time = 1, 3, and 6 h, Chenggong station obtained minimum errors in scenarios 7, 5, and 6, respectively. At the same lead times, Taitung station obtained minimum errors in scenarios 5, 5, and 6, and Dawu station obtained minimum errors in scenarios 5, 4, and 4, respectively. Lanyu station obtained the minimum error in scenario 5 for lead time = 1, 3, and 6 h. Compared with the onshore stations (Chenggong, Taitung, and Dawu), the offshore station (Lanyu) obtained optimal results for one scenario at all three lead times. This may have occurred because the offshore station does not have terrain blockage. Therefore, one radar elevation angle can be used to obtain optimal prediction results in different forecast horizons. Figure 6e–h illustrates the validation results of the XGB prediction model, and Figure 6i–l presents the validation results of the MLR prediction model. Among GRU, XGB, and MLR, the prediction errors obtained in XGB and MLR were generally higher than those obtained in the GRU.

**Figure 7:**
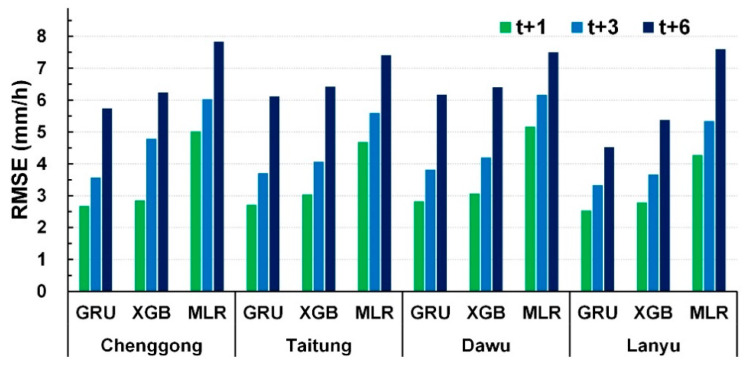
Comparison of RMSE values of GRU, XGB, and MLR prediction models (average results of 10 scenarios).

For comparisons of GRU, XGB and MLR models, Figure 7 averages the results of the 10 scenarios at each station. The GRU prediction model was considerably more effective than the XGB and MLR models were at each station with lead times of 1, 3, and 6 h. Therefore, the GRU model was used in the subsequent analysis. In addition, Lanyu station had smaller RMSEs than Chenggong, Taitung, and Dawu stations did.

## 5. Simulation and Comparison

**Figure 8:**
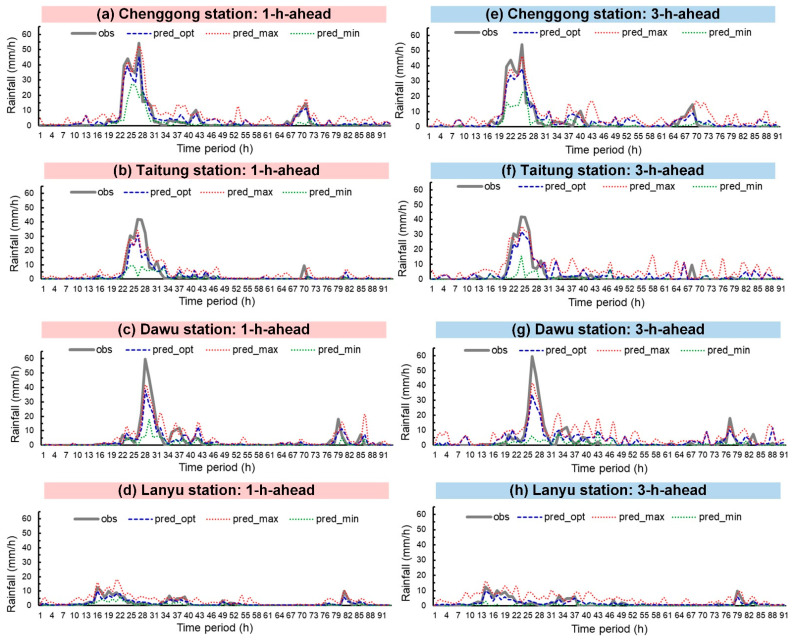
Typhoon Nepartak prediction results: (**a**–**d**) lead time = 1 h; (**e**–**h**) lead time = 3 h.

**Figure 9:**
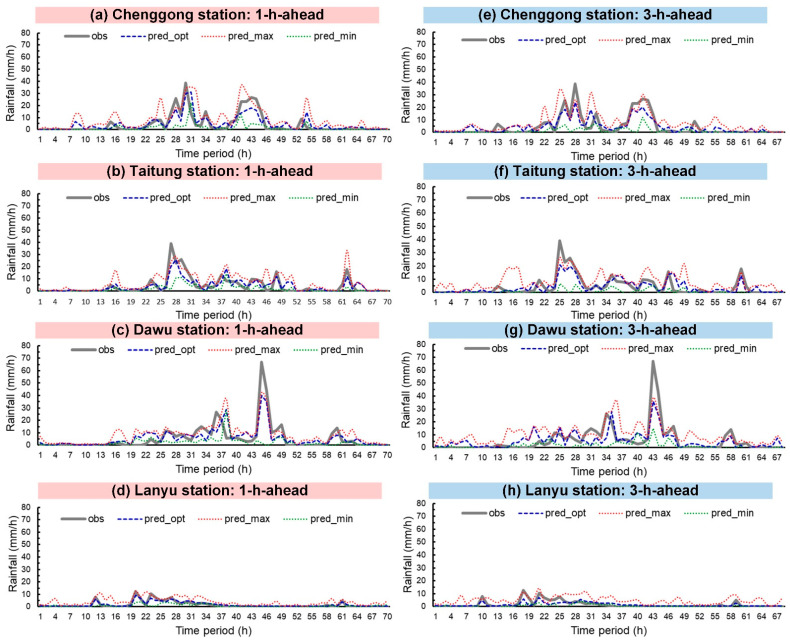
Typhoon Meranti prediction results: (**a**–**d**) lead time = 1 h; (**e**–**h**) lead time = 3 h.

**Figure 10:**
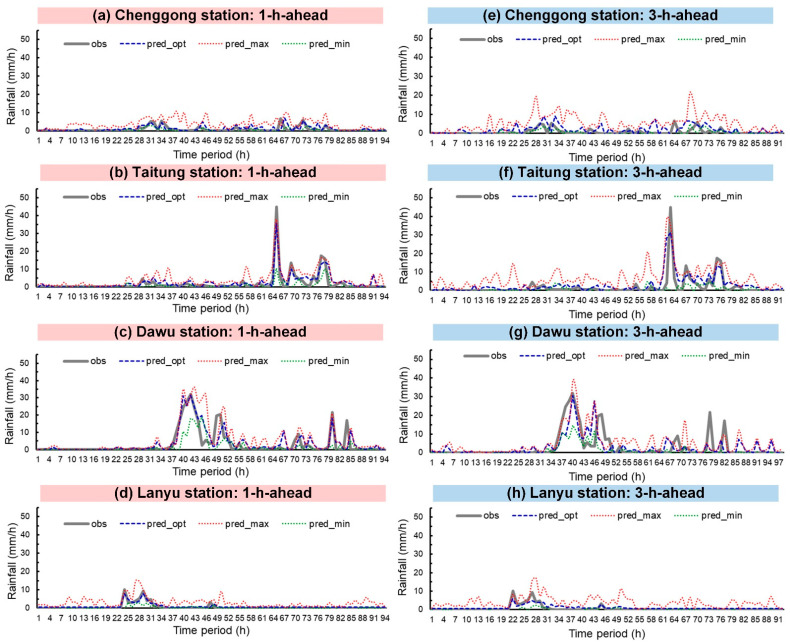
Typhoon Megi prediction results: (**a**–**d**) lead time = 1 h; (**e**–**h**) lead time = 3 h.

Typhoons Nepartak, Meranti, and Megi were simulated. Figure 8, Figure 9 and Figure 10 plot the rainfall forecast results for the three typhoons (lead time = 1 and 3). In the figures, the solid gray line denotes observed rainfall (obs). The real-time forecasts included pred_opt (blue dashed line), pred_max (red dotted line), and pred_min (green dotted line). Hyetograph definitions for pred_opt, pred_max, and pred_min were discussed in Section 3.

As shown in the figures, Chenggong, Taitung, and Dawu stations recorded the maximum observed hourly rainfall for all three typhoons. Typhoon Nepartak reached the heaviest observed rainfall (59.5 mm/h) at Dawu station at time period = 28 h (Figure 8c); Typhoon Meranti attained the heaviest observed rainfall (67.0 mm/h) at Dawu station at 45 h (Figure 9c), and Typhoon Megi reached the heaviest observed rainfall (65.0 mm/h) at Taitung station at 45 h (Figure 10b).

Regarding prediction performance, the graphs indicate that pred_opt was between pred_max and pred_min, as expected. The following comparisons between the three forecasts and obs can be drawn visually: (1) pred_opt predicted future rainfall trends with fluctuations in obs, (2) pred_max generally overestimated lighter rainfall, and (3) pred_min generally underestimated heavy rainfall.

### 5.1. Evaluation Indices of Each Typhoon

**Figure 11:**
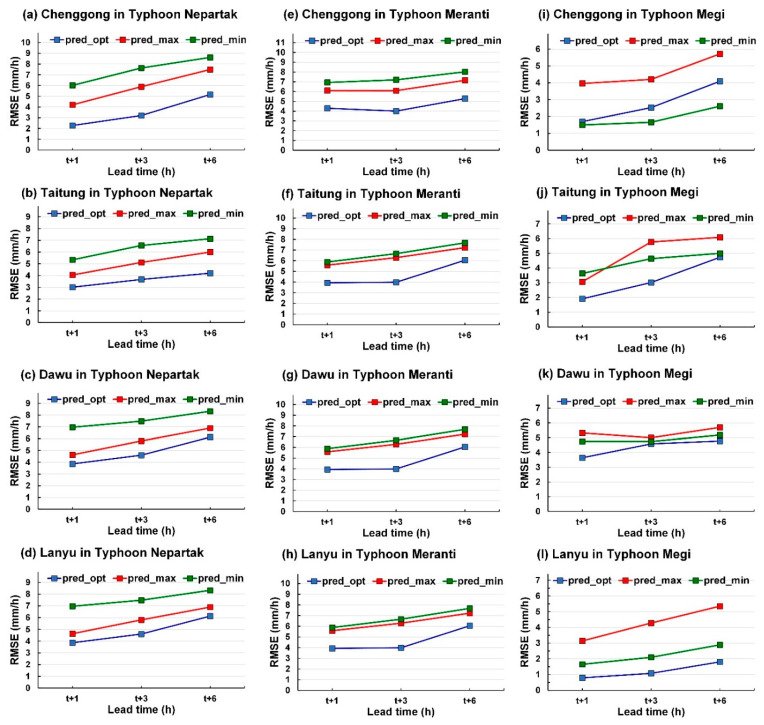
RMSE results for pred_opt, pred_max, and pred_min for each typhoon and station: (**a**–**d**) Typhoon Nepartak; (**e**–**h**) Typhoon Meranti; (**i**–**l**) Typhoon Megi.

To understand the differences between the predicted values (i.e., pred_opt, pred_max, and pred_min) and obs, the RMSEs of each typhoon at lead time = 1, 3, and 6 h were analyzed. As illustrated in Figure 11, the prediction results of pred_opt of the three typhoons were more satisfactory than those of pred_max and pred_min were. Therefore, from a user’s perspective, a theoretically favorable prediction value can be obtained using pred_opt because the 10 scenarios can be used in real-time prediction. In addition, pred_max and pred_min can be used to understand the maximum error that may occur when predicting high and low values and provide users with more prediction information.

### 5.2. Overall Evaluation Index

The averaged evaluation indices for all test typhoons were used to understand prediction errors among stations. Because Chenggong, Taitung, and Dawu stations experienced heavier rainfall than Lanyu station did, the model predictions had higher absolute errors. For prediction, this study used relative RMSE (rRMSE), the equation for which is

(3) $$\mathrm{rRMSE}=\frac{\mathrm{RMSE}}{{\bar{P}}_{}^{\mathrm{obs}}}$$

where ${\bar{P}}_{}^{\mathrm{obs}}$ is the average of observations.

**Figure 12:**
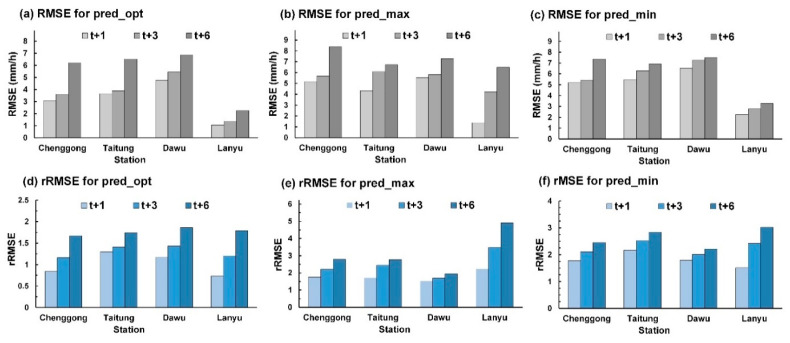
Average RMSE and rRMSE for test typhoons 1, 3, and 6 h ahead: (**a**) RMSE for pred_opt; (**b**) RMSE for pred_max; (**c**) RMSE for pred_min; (**d**) rRMSE for pred_opt; (**e**) rRMSE for pred_max; (**f**) rMSE for pred_min.

Figure 12 plots the average RMSE and rRMSE for all test typhoons. Figure 12a plots the absolute RMSE performance of pred_opt at the four stations, indicating that (1) for onshore stations, Chenggong station exhibited higher performance than Taitung and Dawu stations did and (2) Lanyu station performed far lower than Chenggong, Taitung, and Dawu stations did. Figure 12b presents the rRMSE performance of pred_opt at the four stations, among which (1) for onshore stations, Chenggong station outperformed Taitung and Dawu stations, (2) Lanyu station outperformed Taitung and Dawu stations, and (3) Lanyu station performed more favorably at lead time = 1 h, but less favorably at lead time = 3 and 6 h than Chenggong station did.

### 5.3. Comparison of Predicted and CWB Values

**Figure 13:**
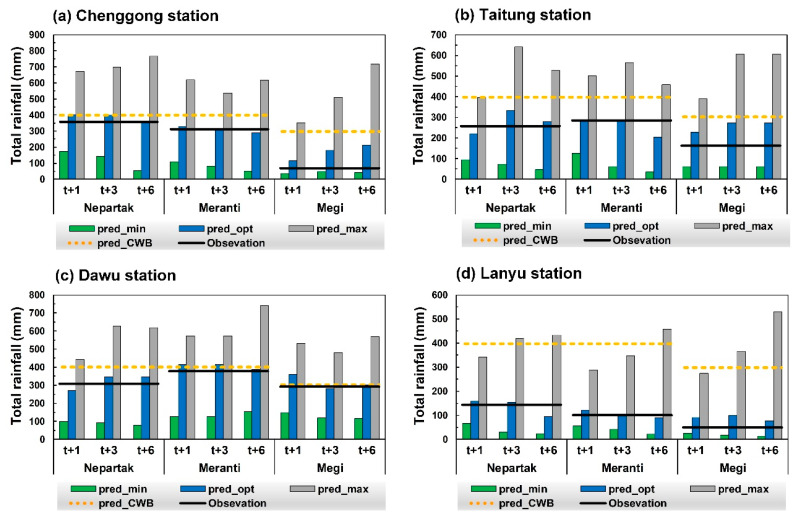
Comparison of observed and predicted total rainfall of test typhoons at each station with CWB nowcasting values: (**a**) Chenggong; (**b**) Taitung; (**c**) Dawu; (**d**) Lanyu.

Nowcasting data for Typhoons Nepartak, Meranti, and Megi released by CWB were compared with the prediction results of this research model to confirm the practicability of the developed conceptual system. Figure 13 plots total obs (solid black line), predicted values (pred_opt, pred_min, and pred_max; histogram), and CWB nowcasting values (pred_CWB; orange dotted line) of the test typhoons at each station. The observations were between pred_min and pred_max. Furthermore, pred_opt was closer to the observation value than pred_CWB was. Therefore, the simulation results of pred_opt have reference value.

This study used the error rate of cumulative rainfall (ERROR_RATE) to evaluate the percentage of the total rainfall prediction error. ERROR_RATE was used to evaluate an average ratio of the absolute errors of cumulative rainfall and was defined as

(4) $$\mathrm{ERROR}\_\mathrm{RATE}=\frac{\sum_{k=1}^{K}\left|R_{k}^{\mathrm{pre}}-R_{k}^{\mathrm{obs}}\right|}{\sum_{k=1}^{K}R_{k}^{\mathrm{obs}}}$$

where $R_{k}^{\mathrm{pre}}$ is the estimated cumulative rainfall for typhoon event *k*; $R_{k}^{\mathrm{obs}}$ is the observed cumulative rainfall for typhoon event *k*; and *K* is the number of test typhoons.

**Figure 14:**
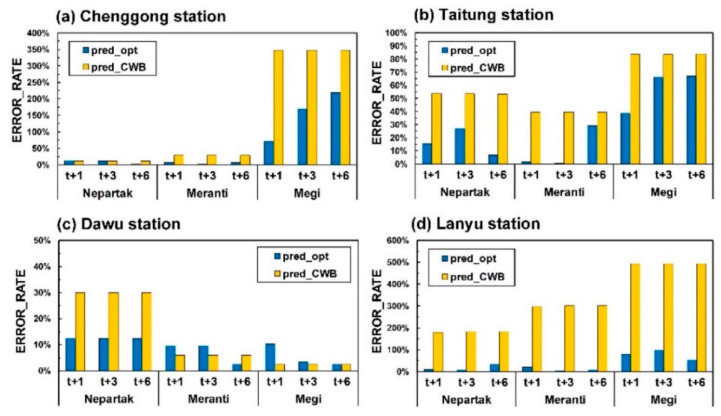
Comparison of ERROR_RATE of pred_opt and pred_CWB: (**a**) Chenggong; (**b**) Taitung; (**c**) Dawu; (**d**) Lanyu.

Figure 14 compares the ERROR_RATE of pred_opt with that of pred_CWB. According to the figure, pred_CWB was more likely to be overestimated than pred_opt was. The average ERROR_RATE values of pred_opt and pred_CWB for the test typhoons were 17.8% and 49.7%, at Chenggong station, 11.2% and 54.8% at Taitung station, 6.3% and 12.4% at Dawu station, and 12.9% and 276.3% Lanyu station, respectively. ERROR_RATE for pred_opt was the lowest for Dawu station, followed by Taitung, Lanyu, and Chenggong stations; for pred_CWB, ERROR_RATE was lowest at Dawu station, followed by Chenggong, Taitung, and Lanyu stations.

## 6. Discussion

This study was conducted in southeastern Taiwan and included three onshore weather stations (Chenggong, Taitung, and Dawu) and one offshore weather station (Lanyu). The Chenggong station exhibited superior prediction results to those of Taitung and Dawu stations. This may have occurred because Chenggong station is closer to the Hualien radar station and experienced less interference caused by topographic factors than Taitung and Dawu stations did. Therefore, Chenggong station may have obtained a higher-quality radar reflectivity signal. In addition, the rRMSE results indicated that although radar reflectivity signals at Lanyu station were less affected by topographic factors, reflectivity quality may have been slightly affected because the station was farthest from the Hualien radar station; however, it outperformed Taitung and Dawu stations.

Regarding the overshooting of the beam and blockage, the radar elevation angles derived from the model-induced results were examined. The Chenggong station is possibly sheltered by the Coastal Mountain Range (an average height of 1000 m; Figure 1). The theoretical elevation angles can be computed (Figure 1). The height of the cloud top in a typhoon is assumed to be 18 km according to [76]. The distance from the Hualien radar station to the Chenggong station is 102 km and to the northern tip of the Coastal Mountain Range is 15 km. The elevation of Hualien radar station is 53 m. Therefore, the range of radar elevation angles is from 3.61° to 10.01°. According to Section 4, the optimal results from the GRU model can be derived in scenarios 7, 5, and 6 at lead time = 1, 3, and 6 h, respectively. The corresponding elevation angles are 9.9°, 4.3°, and 6.0°, which reveal that the model results appear reasonable because they fall in the range of theoretical elevation angles.

The Taitung and Dawu stations seem mainly affected by the East Rift Valley (an average height of 250 m; Figure 1). The Hualien radar station is 146 and 196 km away from the Taitung and Dawu stations, respectively, and the northern tip of the East Rift Valley is 30 km away. Therefore, the range of radar elevation angles is 0.38° to 7.05°. The verification results revealed the optimal elevation angles are at 4.3°, 6.0°, and 6.0° (corresponding to scenarios 5, 5, and 6, respectively) at lead time = 1, 3, and 6 h for the Taitung station and 4.3°, 3.4° and 3.4° (scenarios 5, 4, and 4, respectively) for the Dawu station, which demonstrated these were reasonable results. Finally, the Lanyu station possibly is not affected by terrain blockage. The Hualien radar station is 218 km away from the Lanyu station. The theoretical elevation angles can be from 0° to 5.24°. The verification results revealed the optimal elevation angles are all at 4.3° at lead times 1, 3, and 6 h. Therefore, the most suitable elevation angles can be successfully identified.

## 7. Conclusions

This study developed a real-time rainfall forecasting system that can predict rainfall in an area a few hours before a typhoon arrives at Taiwan. To effectively predict rainfall data, this study used reflectivity data obtained from nine elevation angles in the Taiwan Doppler VCP21 system and ground-weather data in southeastern Taiwan. The area included three onshore weather stations (Chenggong, Taitung and Dawu) and one offshore station (Lanyu). Radar data were provided by the Hualien WSR. This study analyzed 14 typhoons that affected the study area from 2008 to 2017. GRU was used to establish the prediction model, and XGB and MLR were used as the benchmark. The GRU prediction model was substantially superior to the XGB and MLR model in forecasting rainfall at lead times of 1, 3, and 6 h at each station.

The simulation results for three test typhoons revealed that the predictive performance of pred_opt was more satisfactory than that of pred_max or pred_min was. Therefore, when developing a real-time prediction system, 10 scenarios can be used to select an optimal solution (i.e., pred_opt) to obtain a favorable prediction value. In addition, pred_max and pred_min can be obtained to provide the probable ranges of predicted values. Consequently, the overall results revealed that the input data set combined with weather-station data and radar reflectivity at the optimal elevation angle yielded optimal results for short-term rainfall forecasting. Moreover, nowcasting values released by the CWB were compared with the predicted values of this study, which demonstrated that pred_opt was closer to the observed values than pred_CWB was. Therefore, the simulation results of the system have practical reliability.

## Figures and Tables

**Table 1:** Typhoon events affecting the study area.

Year.	Date	Typhoon	Precipitation (mm)				Maximum Wind Speed of Typhoon Center (m/s)
			Chenggong	Taitung	Dawu	Lanyu	
2008	07/27~07/28	Fung-Wong	172.8	96.0	56.0	56.0	43
2010	09/19~09/20	Fanapi	273.2	200.4	361.3	64.0	45
2011	08/27~08/30	Nanmadol	360.0	382.5	597.3	373.6	53
2012	08/23~08/28	Tembin	459.0	441.2	464.1	265.8	35
2013	09/21~09/23	Usagi	314.0	245.8	262.0	135.9	55
2014	07/21~07/23	Matmo	393.7	195.0	140.5	412.5	38
2014	09/19~09/21	Fung-Wong	230.6	237.5	365.5	113.5	25
2015	08/07~08/09	Soudelor	159.4	25.8	285.5	35.8	48
2015	08/21~08/22	Goni	140.0	147.8	166.2	342.9	51
2016	07/07~07/10	Nepartak	400.7	265.5	311.5	147.6	58
2016	09/13~09/15	Meranti	309.5	286.5	378.0	103.0	60
2016	09/26~09/29	Megi	67.0	163.4	309.3	81.5	45
2017	07/29~07/31	Nesat	112.0	137.8	338.0	148.4	40
2017	08/21~08/23	Hato	200.0	266.5	265.5	237.5	33

## Data Availability

The related data were provided by Taiwan’s CWB, which are available at https://rdc28.cwb.gov.tw/ (accessed on 1 August 2020).
